# Enhancing the performance of recycled aggregate concrete through optimized pretreatment methods: a microstructural perspective

**DOI:** 10.1038/s41598-025-14834-y

**Published:** 2025-08-16

**Authors:** Maja Kępniak, Filip Chyliński, Piotr Woyciechowski

**Affiliations:** 1 Faculty of Civil Engineering, Warsaw University of Technology, Al. Armii Ludowej 16, 00- 637 Warsaw, Poland; 2 Instytut Techniki Budowlanej, Filtrowa 1, 00-611 Warsaw, Poland

**Keywords:** Recycled aggregate, Interfacial transition zone, Cement hydration, Recycled aggregate pre-treatment, Microstructure, Durability, Civil engineering, Structural materials

## Abstract

Using recycled concrete aggregates (RCA) in new concrete production presents a sustainable solution to construction waste challenges, yet the inherent weaknesses of the interfacial transition zone (ITZ) between old and new cement matrices remain a critical limitation. This study evaluates three industrially feasible pretreatment methods—sieve-washing, water immersion, and resin impregnation - through a novel combination of semi-quantitative microstructural analysis and durability testing. Our findings demonstrate that resin treatment reduces carbonation depth by 38% compared to untreated RCA, primarily through pore structure modification. At the same time, water immersion enhances ITZ density by optimizing moisture release during hydration. However, resin-treated aggregates exhibited significantly lower compressive strength due to poor adhesion of the new paste. After 28 days, concrete with saturated recycled aggregate showed similar compressive (49.1 ± 0.7 MPa) and flexural strength (5.8 ± 0.3 MPa), but slightly higher carbonation depth than the concrete with only natural aggregate. The calcium concentration at a distance from the recycled grain indicates that saturated and resin pre-treatment influence the ITZ’s density best. The research establishes a practical framework for selecting pretreatment methods based on measurable ITZ characteristics and project-specific durability requirements, advancing academic understanding and practical implementation of sustainable concrete technologies.

## Introduction

In light of the imperative of implementing principles of sustainable development, including waste management and a circular economy, the utilization of recycled aggregate becomes increasingly significant due to its potential environmental and economic benefits.

Incorporating recycled aggregate (RA) in construction offers significant environmental advantages. Reducing reliance on waste landfills and minimizing the depletion of natural aggregates promotes resource conservation and waste reduction in the construction industry^1,2^. Moreover, the reuse and recycling of construction and demolition waste, including recycled aggregate, contribute to mitigating environmental impacts associated with new building projects^3^.

Unfortunately, the utilization of recycled aggregate without proper preparation can lead to the following effects: increases of the content of recycled aggregate in the whole aggregate mix have resulted in worsened workability^4^, decreased bulk density^5^, reduced permeability^6^, diminished mechanical strength^7^, and lowered modulus of elasticity^8^. Additionally, intensified bleeding^9^, increased shrinkage^10^, and enhanced creep^11^ have been noted. These alterations in concrete properties are attributed to adhered mortar in recycled aggregate.

Therefore, effective pre-treatment of recycled aggregates is essential for enhancing their properties and performance in concrete structures^12^. Various techniques, such as removal of adhered mortar, polymer impregnation, and pozzolanic paste immersion, have been identified to improve mechanical strength and durability in recycled aggregate concrete (RAC)^9^. Pre-saturation of recycled aggregates before mixing addresses consistency issues and enhances concrete properties, further emphasizing the importance of pre-treatment in optimizing performance^13^. This method facilitates the cleaning of RA and enhances its adhesion to the cementitious paste. However, determining the effective water content remains problematic. It appears that the compressive strength is significantly influenced by how the water absorption of RA is considered in the mix design, and full compensation of water absorption by aggregates is not recommended^14^. This suggests that water absorbed by the aggregate will remain in the aggregate longer than cement hydration occurs. It will only be released in later stages of curing, contributing to the moisture retention in the concrete^15^.

Pre-treated recycled aggregate presents favorable economic implications, including cost savings^1^. Studies indicate that utilizing recycled aggregates as non-structural materials can lead to significant cost reductions compared to traditional materials^16,17^. These economic benefits underscore the potential of recycled aggregate as a sustainable alternative in construction projects.

Despite its environmental and economic advantages, the widespread adoption of recycled aggregate faces challenges and limitations^18^. The inferior characteristics of recycled aggregates and non-uniformity in mixed compositions pose hurdles to their structural use. While pre-soaking and treatment techniques can improve specific properties, addressing these inherent limitations remains crucial for promoting the broader acceptance of recycled aggregate in structural materials^19^.

The quest for effective RCA pretreatment has generated diverse technological approaches, each with distinct mechanisms and implementation challenges. Ball milling techniques achieve up to 95% attached mortar removal through abrasive mechanical action, while acid treatments using hydrochloric or sulfuric solutions chemically dissolve cementitious residues^20,21^. Microwave-assisted processing leverages differential thermal expansion to separate aggregates from mortar, and bio-deposition methods employ microbial calcium carbonate precipitation to seal surface pores^22,23^. Our selection of sieve-washing, water immersion, and resin impregnation stems from a critical evaluation of scalability and environmental impact. While advanced methods show superior mortar removal rates in laboratory settings, their energy demands often exceed 2.5 kWh/tonne, prohibitively high for large-scale operations^24,25^. Chemical treatments introduce secondary pollution risks from residual acids or bioreagents, complicating wastewater management. The chosen methods balance effectiveness with practical feasibility, requiring neither specialized equipment nor hazardous material handling, making them adaptable to conventional concrete batching plants^4^.

**Fig. 1:**
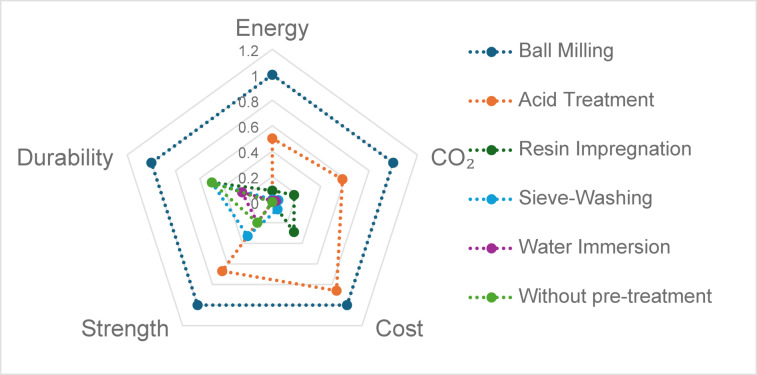
Normalized performance comparison of pre-treatment methods for recycled aggregates according to the data from^26–33^.

The environmental footprint of RCA pretreatment is a growing concern. Despite its higher material cost, resin impregnation offers a relatively low carbon footprint compared to thermochemical methods, as it avoids high energy consumption and hazardous byproducts. Sieve-washing and water immersion require minimal infrastructure and energy, reducing environmental impact. A comparative relative evaluation of energy consumption, CO₂ emissions, and implementation costs for various methods is provided in Table 1; Fig. 1, highlighting the sustainability advantages of the selected approaches.

**Table 1:** Comparison of energy consumption, carbon footprint, and implementation cost of recycled aggregate pretreatment methods according to the data from^26–33^.

Method	Energy Consumption (kWh/tonne)	CO₂ Footprint (kg/tonne)	Implementation Cost ($/tonne)
Ball Milling	8.2	6.5	15–20
Acid Treatment	4.1	3.8*	12–18
Resin Impregnation	0.7	1.2	4–6
Sieve-Washing	0.2	0.3	0.8–1.2
Water Immersion	0.1	0.2	0.6–1.0

*Includes neutralization process emissions.

One of the key aspects in shaping concrete with recycled aggregate is ensuring the adequate formation of the Interfacial Transition Zone (ITZ). In conventional concrete, there is only one Interfacial Transition Zone (ITZ), which has a thickness of up to approximately 100 micrometers^34,35^. Due to the formation of calcium hydroxide (CH) crystals on the surface of aggregate particles and the local increase in the water-cement ratio, a more porous volume of the paste is created^36,37^. This region is generally weaker than the rest of the paste volume. Consequently, it is the lowest zone in cement concrete in terms of strength and durability^38,39^.

More diverse ITZ zones are present in the case of concrete with recycled aggregate. In addition to the ITZ between the new paste and natural aggregate, often a Recycled Aggregate Concrete (RAC) component, the following zones also exist: Firstly, there is an ITZ between the paste and the aggregate within the recycled aggregate particles. Secondly, there is a transition zone between the new paste and the surface of the old paste, as well as an ITZ between the surface of the aggregate in the recycled aggregate particle and the new paste^40^. Therefore, the problem is complex.

The research investigated how different scenarios of preparing recycled aggregate affect the formation of the interfacial transition zone (ITZ) between recycled aggregate particles and the new cement paste. The study primarily analyzed various preparation methods of the aggregate, resulting in different levels of internal moisture content within the recycled aggregate particles and on their surface and varying levels of acceptable particulate content. Additionally, the research examined how changes in the structure of the ITZ influence the compressive and flexural strength, as well as the carbonation resistance of the resulting concrete.

## Materials

Concrete mixes with a fixed quantitative composition were designed as part of the work. They differed qualitatively in the method of preparing the recycled aggregate. The composition of the mixes was as follows: Portland Cement CEM I 42.5 R (310 kg/m³), water (100 kg/m³), river sand 0/2 N (600 kg/m³), gravel fraction 2/4 N (400 kg/m³), and recycled aggregate fraction 4/8R (500 kg/m³). Two fractions of natural gravel aggregate were used: 2/4 (designated as 2/4 N) and 4/8 (designated as 4/8 N). The fineness modulus of the coarse natural aggregates is 5.56 and 5.63, respectively (Table 2). The analyzed 4/8 fraction of recycled aggregate (designated as 4/8R) originated from demolishing a residential building. Consequently, the composition of the recycled aggregate is varied. The dominant component is cement concrete—Rc (88.5%) and unbound aggregate Ru (4.7%). The composition of recycled aggregate also included bituminous material Ra (1.9%), clay elements, and autoclaved aerated concrete elements Rb (0.2%). The fines (fraction 0/0.063) content was 0.5%. Since the mixes were prepared with recycled aggregate of varying moisture content, the actual dosing of the components varied. A lignosulfonate and gluconate-containing admixture was dosed up to achieve a constant consistency. Based on preliminary studies, it was assumed that half of the water in the recycled aggregate is part of the effective water content. The assumption that 50% of RCA pore water contributes to adequate mixing water derives from rheological observations. Mixes with 24-hour pre-soaked aggregates demonstrated equivalent slump (120 ± 5 mm) to control mixes when total water was reduced by 48–52%, indicating partial moisture release during mixing (Table 3).

**Table 2:** Basic characteristics of the used aggregates.

Aggregate	0/2 *N*	2/4 *N*	4/8 *N*	4/8R
Density (EN 1097-6), g/cm³	2.54	2.64	2.67	2.53
Dry density (EN 1097-6), g/cm³	2.13	2.46	2.60	2.25
Water absorption (EN 1097-6), %	7.5	2.80	1.20	10.0
Los Angeles coefficient (EN 1097-2), %	-	27.0	25.0	75.0
Fines (fraction 0/0.063) content (EN 933-1), %	1.0	0.1	0.1	0.5

**Fig. 2:**
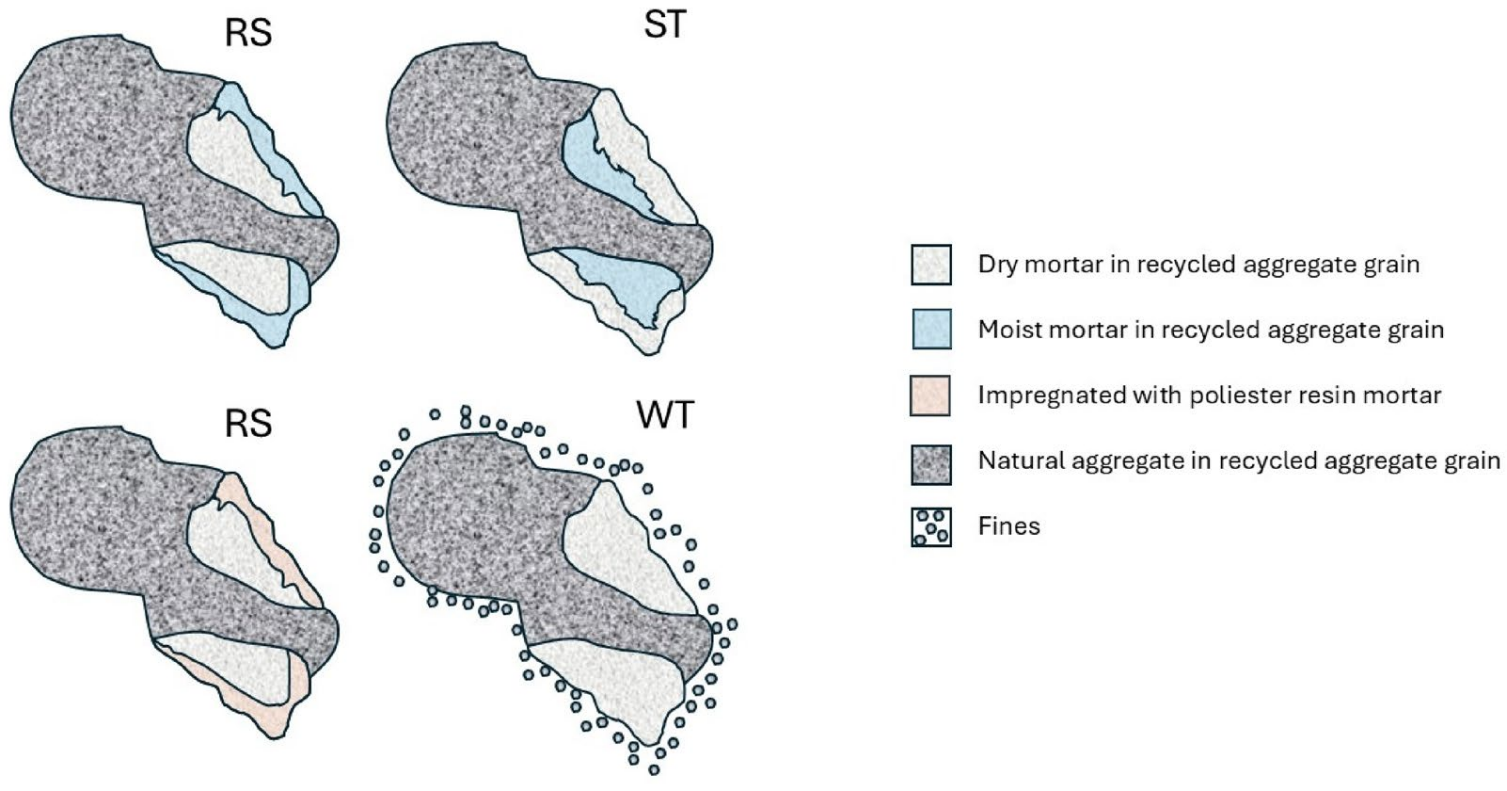
View of recycled aggregate grain after selected preparation treatments (schematic representation).

The scenarios for preparing recycled aggregate were as follows (Fig. 2):

- WT - without treatment (air dry): The aggregate was assumed to contain fines and was completely dry when dosing into the concrete mix.
- RS - Rinsed Saturated: The aggregate was rinsed on a sieve with dimensions of 0.063 mm, and then directly dosed into the concrete mix. It was assumed to be free of fines, with water mainly present on the surface of the aggregate particles.
- ST - Soak treatment: The recycled aggregate was soaked in water for 24 h, then surface dried before dosing into the concrete mix. Water was assumed to be predominantly within the interior of the aggregate particles, with some fines removed but not entirely.
- RT - Resin treatment: After rinsing and drying to a constant mass, the aggregate was subjected to surface impregnation with a synthetic vinyl-ester resin of low viscosity (350 ± 50 mPa·s at 25 °C). This treatment aimed to eliminate fines and create a surface barrier preventing water from penetrating the interior of the aggregate particles. The resin also reinforced the aggregate by penetrating its porous structure.

A mix without recycled aggregate, designated as R (reference), was also prepared as a reference for the concrete properties.

**Table 3:** Concrete mixtures composition for one cubic meter, R – reference without recycled aggregate, WT – without treatment, RS – rinsed saturated, ST – Soak treatment, RT – resin treatment.

	*R*	WT	RS	RT	ST
Portland Cement CEM I 42,5, kg	440	440	440	440	440
Water, kg	220	220	176	220	176
Recycled aggregate water content, %	0	0	10	0	10
Effective water content, kg	220	220	220	220	220
Recycled aggregate,4/8R, kg	-	858	902	858	902
Gravel 2/4 N, kg	430	430	430	430	430
Gravel 4/8 N, kg	858	-	-	-	-
River sand 0/2 N, kg	858	858	858	858	858
Admixture, % cement mass	1.0	3.0	2.0	1.0	2.0

**Fig. 3:**
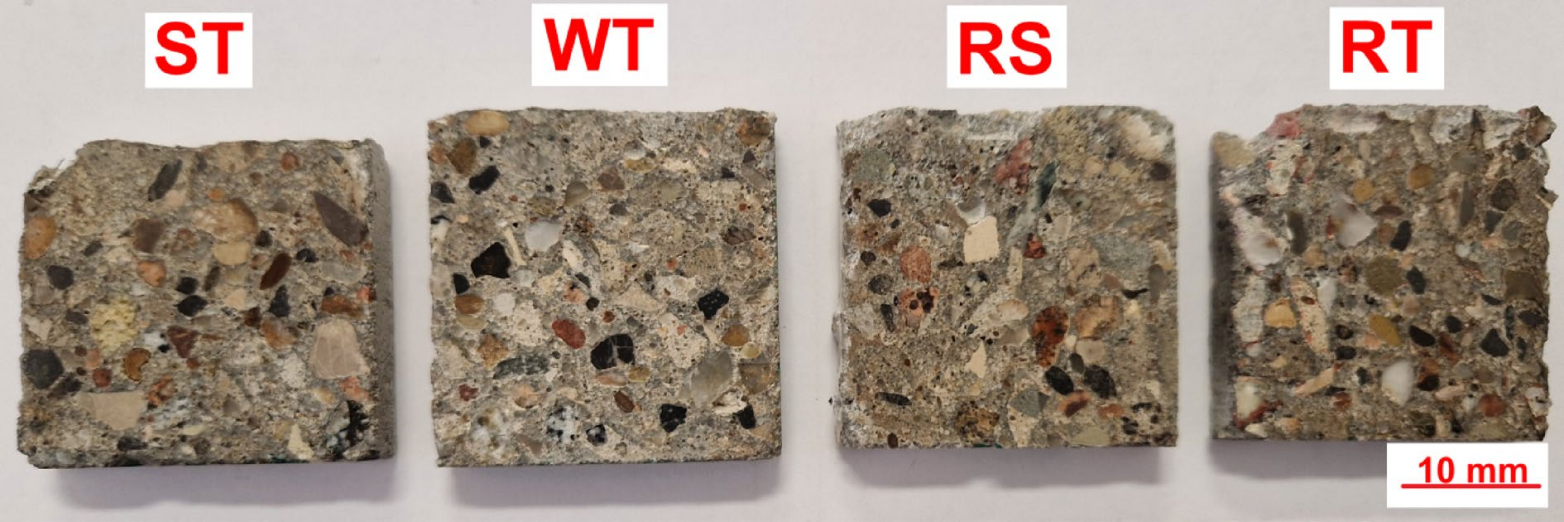
Polished sections prepared for SEM analysis.

Microstructural investigations of concrete aim to assess the microstructure of concrete with the addition of recycled concrete aggregate in various states of water saturation and fines content. In addition, the microstructure of the aggregate-grout contact zone was analyzed to determine the causes of the observed changes in the strength properties of concrete with the addition of recycled concrete aggregate with various degrees of water saturation and different fines content (rinsed and non-rinsed). Several tests were carried out on the elemental composition of the grout areas using the SEM-EDX technique at various distances from the aggregate grains to assess changes in the concentration of the main components and changes in the contact zone. Figure 3 shows samples of WT, RT, ST, and RS concrete sections prepared for SEM analysis.

**Fig. 4:**
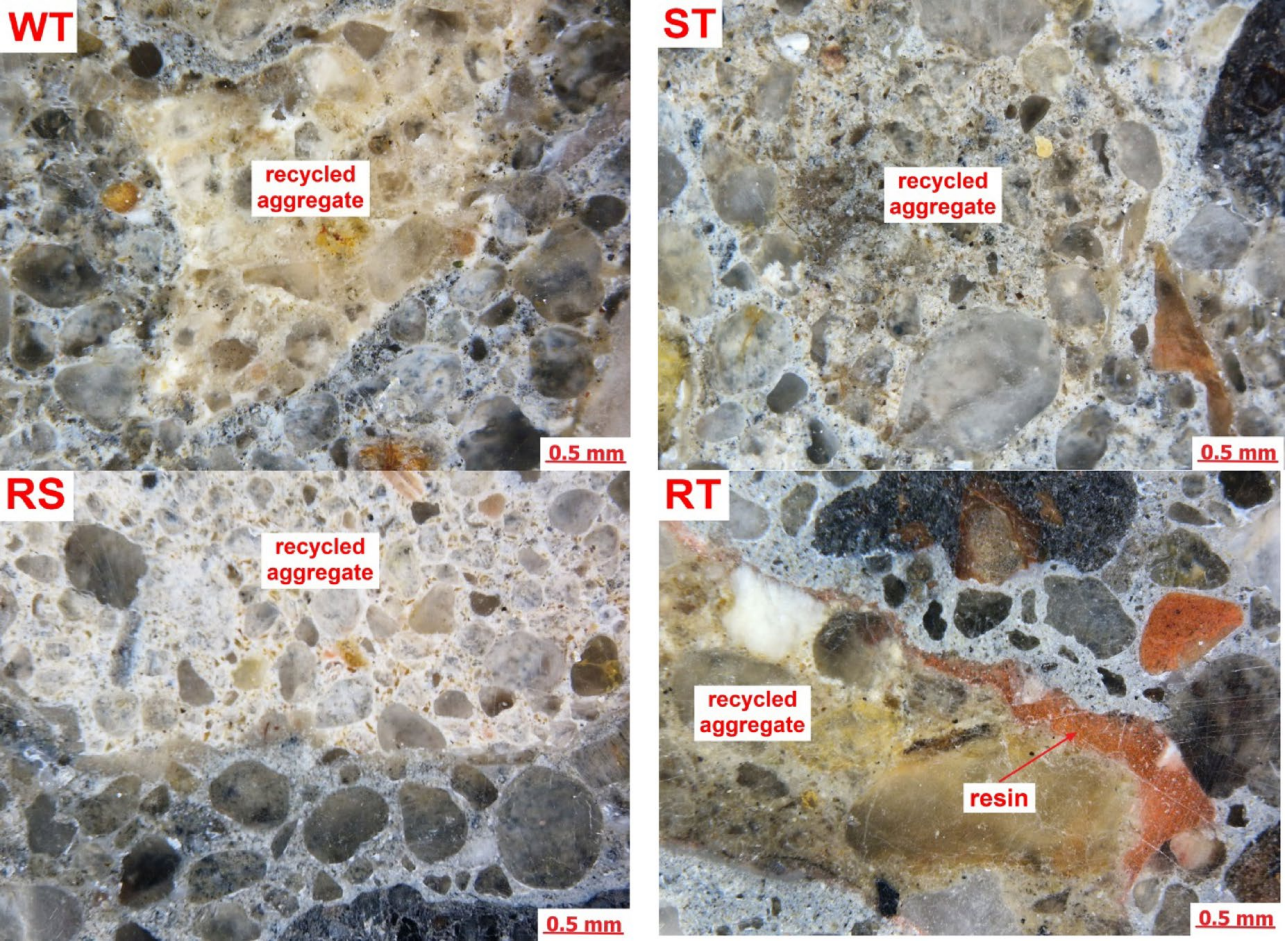
Microstructure of polished samples of concrete – optical microscopy.

Figure 4 presents examples of the microstructure of concrete samples examined using optical microscopy. Optical microscopy observations aimed to select areas of interest for SEM analysis.

## Methods

Microstructural examinations were carried out on polished sections of concretes in two stages – first using an optical microscope and second using a scanning electron microscope (SEM).

The microstructural analysis protocol was rigorously optimized to ensure representative characterization of ITZ features. Samples underwent 24-hour preparation sequences:

- Resin impregnation: Epoxy infiltration under 0.5 bar vacuum pressure for 6 h.
- Grinding/polishing: Progressive abrasion from 120-grit to 3000-grit silicon carbide papers.
- Conductive coating: 15 nm gold-palladium sputter coating at 20 mA for 120 s.

This standardized procedure, validated through parallel testing with untreated specimens, minimizes artificial pore formation while preserving original ITZ morphology. The extended impregnation duration ensures complete resin penetration through RCA pore networks, which is critical for accurate void structure analysis.

A stereoscopic optical microscope produced by ZEISS, model Stemi 508, examined reflected light. The Sigma 500 VP produced by ZEISS (Carl Zeiss Microscopy GmbH, Köln, Germany) was used as an SEM microscope model. Analysis of microstructure was performed using a BSD (backscattered electron detector). Analysis of composition in the micro-area was conducted using an EDX detector produced by Oxford model Ultim Max 40 (Oxford Instruments, High Wycombe, UK). Samples were prepared by polishing thin sections cut perpendicularly to the trowelled surface from the middle of each mortar type. Then, samples were soaked in resin, ground, and polished as described in a previous publication^41^ before SEM examinations, and samples were sputtered with gold.

The consistency of the mixture was evaluated following the procedure outlined in the PN-EN 1015-3 standard for measuring the plasticity of construction mortars. Immediately after mixing the ingredients, a truncated cone was formed on the flow table, with dimensions of 100 mm at the base, 70 mm at the top, and a height of 60 mm. The freshly prepared composite was then subjected to 15 consecutive drops by lifting and dropping the measuring table to a height of 10 mm at a rate of one drop per second. The resulting flow diameter was subsequently measured. Three rectangular specimens measuring 40 mm by 40 mm by 160 mm were prepared for flexural strength testing for each composition, following the EN 196-1 standard. The specimens were tested using the three-point loading method. For compressive strength testing, six specimens were prepared for each composition according to the EN 196-1 standard, with a compressive area of 1600 mm². The PN-EN 13,295 standard also assessed the extent of carbonation. Accelerated carbonation testing followed PN-EN 13,295 under controlled conditions: CO₂ concentration: 1.0 ± 0.2% (volume), relative humidity: 60 ± 5%, temperature: 20 ± 2 °C (climate chamber controlled). Phenolphthalein indicator solution (1% ethanol) was applied to split specimens to track carbonation front progression, for a test duration of 28 days.

## Results and discussion

Analysis of the microstructure of concrete samples containing recycled aggregates, which were variously prepared before adding them to the concrete mix, was made. Microstructural analyses focused on the cement matrix near the transition zone between recycled aggregates and grout grains.

### WT - without treatment

**Fig. 5:**
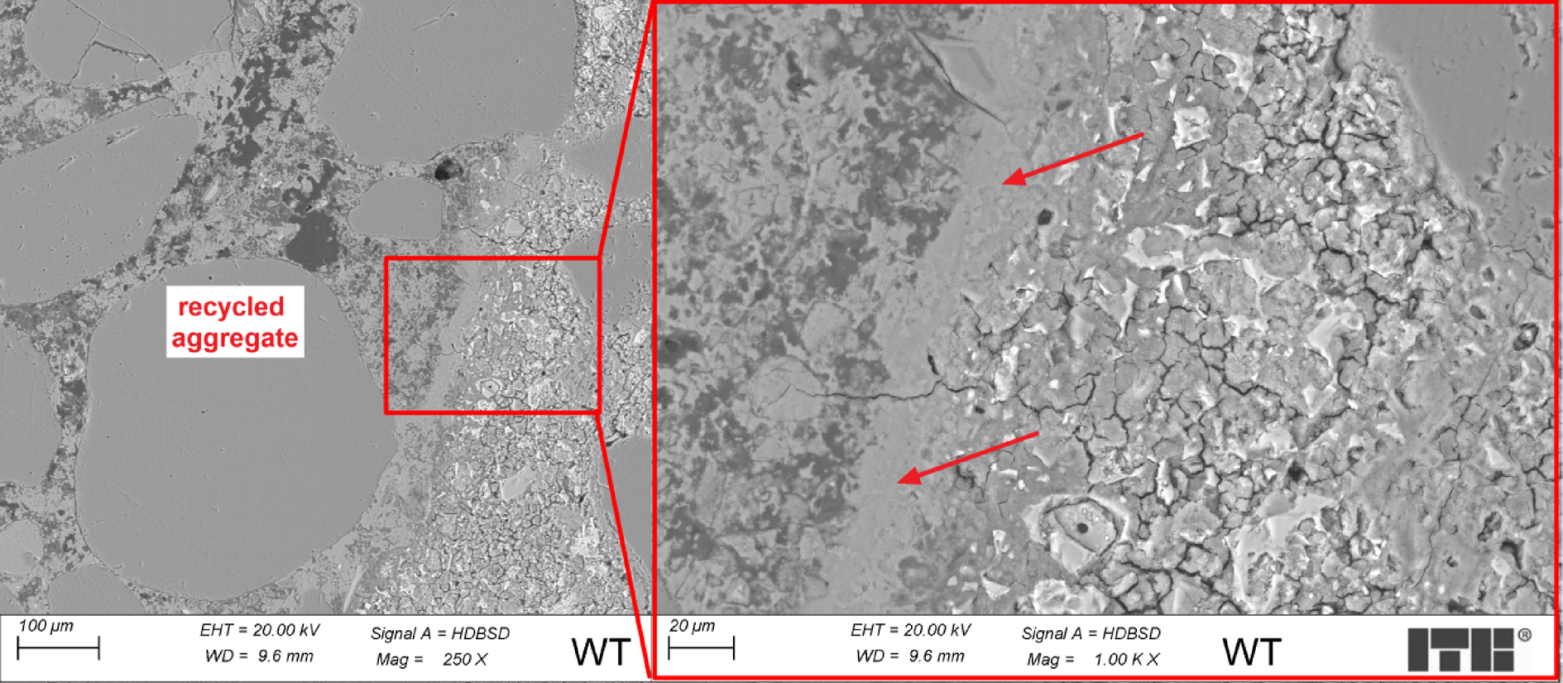
Microstructure of WT concrete sample (arrow marks the transition zone).

Figure 5 presents an example image of the microstructure of a WT concrete sample where recycled aggregates without any treatment have been added to the concrete.

Analyzing the areas of the transition zone between recycled aggregate and cement paste, it has been discovered that the layer of fines has covered areas of the porous surface of the recycled aggregate. The C-S-H phase near the transition zone was sealed with relatively low porosity, mostly continuous, and without excessive cracks. Seal form of C-S-H phase near the transition zone compared to the areas located further might be explained by the absorption of water from the fresh mix by the fines covering recycled aggregates and porous grains. This caused a local decrease in the water-cement matrix, which affects its lower porosity. This phenomenon should affect the increase in concrete’s compressive strength. However, a layer of fines covering some areas of recycled aggregate might affect the lowering of adhesion of the cement matrix to the surface of grains, which will decrease the compressive strength of the composite.

### ST- soak treatment

**Fig. 6:**
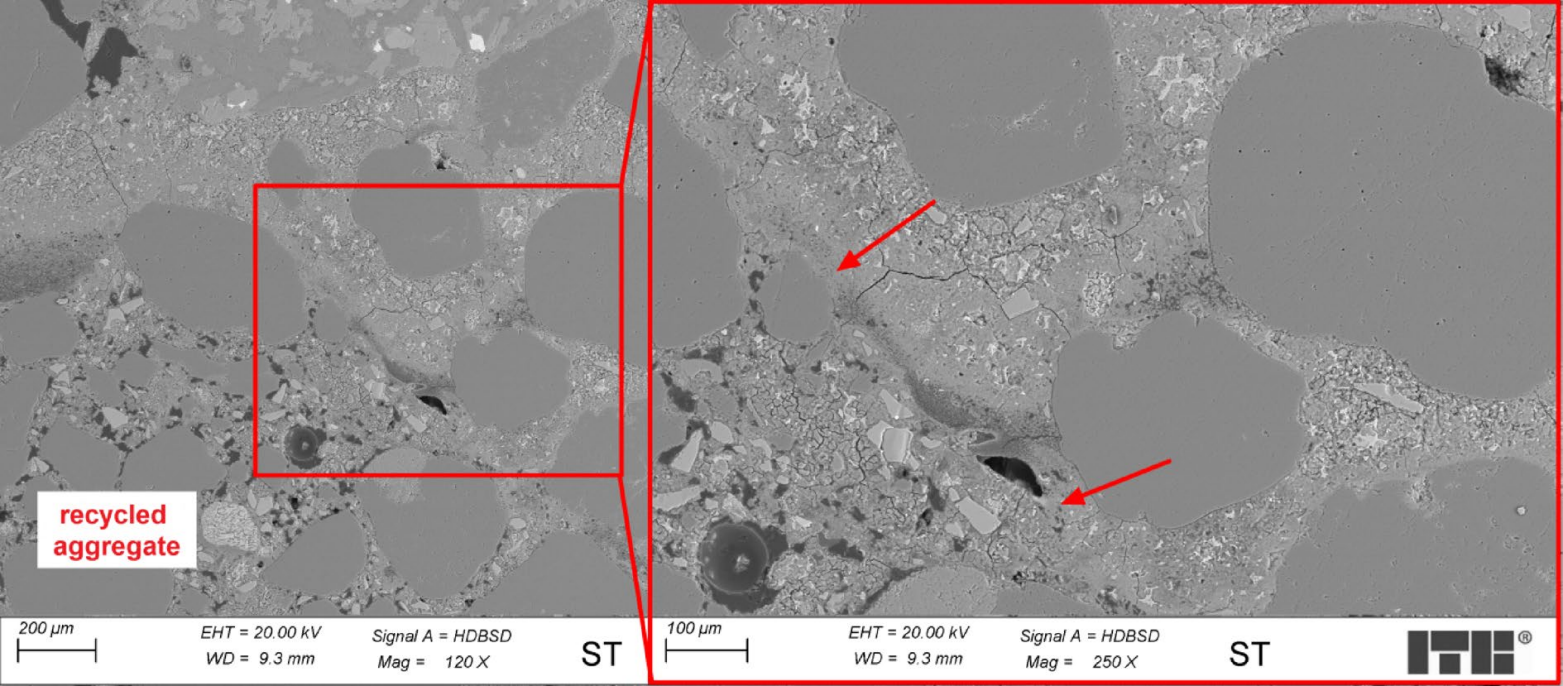
Microstructure of ST concrete sample (arrow marks the transition zone).

Figure 6 presents an example of ST concrete microstructure near the transition zone of recycled aggregate and cement paste.

The transition zone in the ST concrete between grains of recycled aggregate and cement paste differed from that in WT samples. In the transition zone, some discontinuities and small water lenses were observed. C-S-H phase in the areas near the surface of aggregate grains was relatively more porous than in the regions located further. The recycled aggregates in the ST concrete sample were soaked in water until fully saturated before being added to the concrete mix. They were saturated with water and then in a cement matrix, so they couldn’t absorb more water from the concrete mix. The differences between the content of soluble ions in water inside porous aggregate grains and in water in the paste mix might cause water migration outside the aggregate grains. This caused an increase in the water-cement ratio in the areas near the transition zone, which results in higher porosity of the C-S-H phase and, in extreme cases, even the formation of water lenses as observed in Fig. 5.

### RS – rinsed saturated

**Fig. 7:**
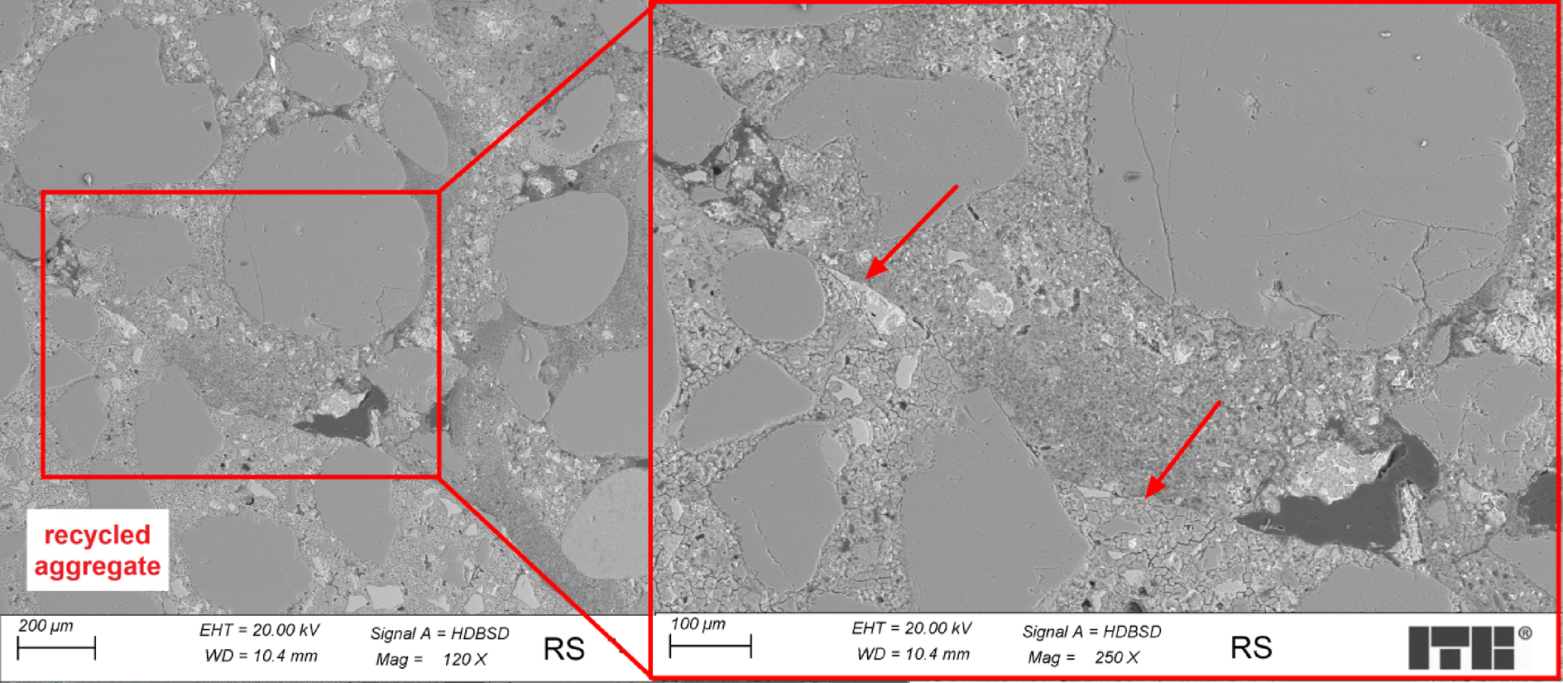
Microstructure of the RS concrete sample (arrow marks the transition zone).

Figure 7 presents an example of the microstructure of RS concrete in the transition zone between recycled aggregate and cement paste.

Analysis of the microstructure of RS concrete samples was focused on the transition zone between recycled aggregate and cement paste. Recycled aggregate, before adding it to the concrete mix, was rinsed with water to remove fine particles, and then it was saturated with water. In this state, the aggregates were added to the concrete mix. Some discontinuities were observed between recycled aggregate and cement paste in the transition zone, and water lenses were larger than in ST concrete samples. C-S-H phase in areas located near the transition zone was more porous than in the regions situated further away. The reason for observed differences in the transition zone relating to the WT concrete samples is the preparation of recycled aggregates before adding them to the concrete mix. Rinsing aggregates with water helps to eliminate the fine particles, which weaken when located in the transition zone. However, it might also cause bigger water lenses observed in the transition zone than in the ST samples. Saturation aggregates with water locally increase the water-cement ratio near the transition zone, making the C-S-H phase more porous.

### RT – resin treatment

**Fig. 8:**
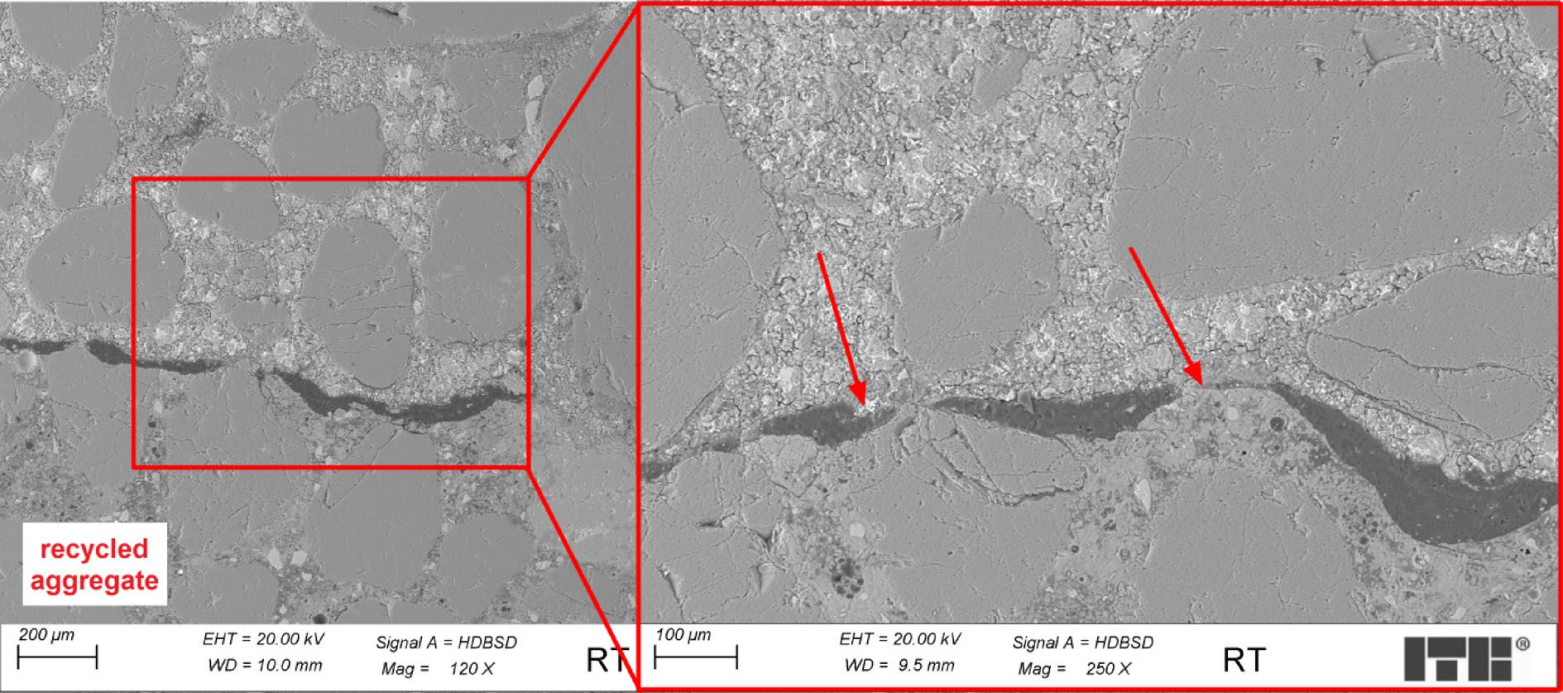
Microstructure of RT concrete sample (arrow marks the transition zone).

Figure 8 presents an example of the microstructure of RT concrete in the transition zone between recycled aggregate and cement paste.

Examinations of the microstructure of the transition zone of RT concrete samples showed that it was continuous, without excessive cracks, and quite sealed. As shown in Fig. 6, dark areas in the transition zone are not water lenses but residue resin left by the preparation of recycled aggregate before adding it to the concrete mix. The C-S-H phase was quite dense, and differences between areas near the transition zone and areas located further in the cement grout were not so noticeable.

### Semi-quantitative analysis of the transition zone

**Fig. 9:**
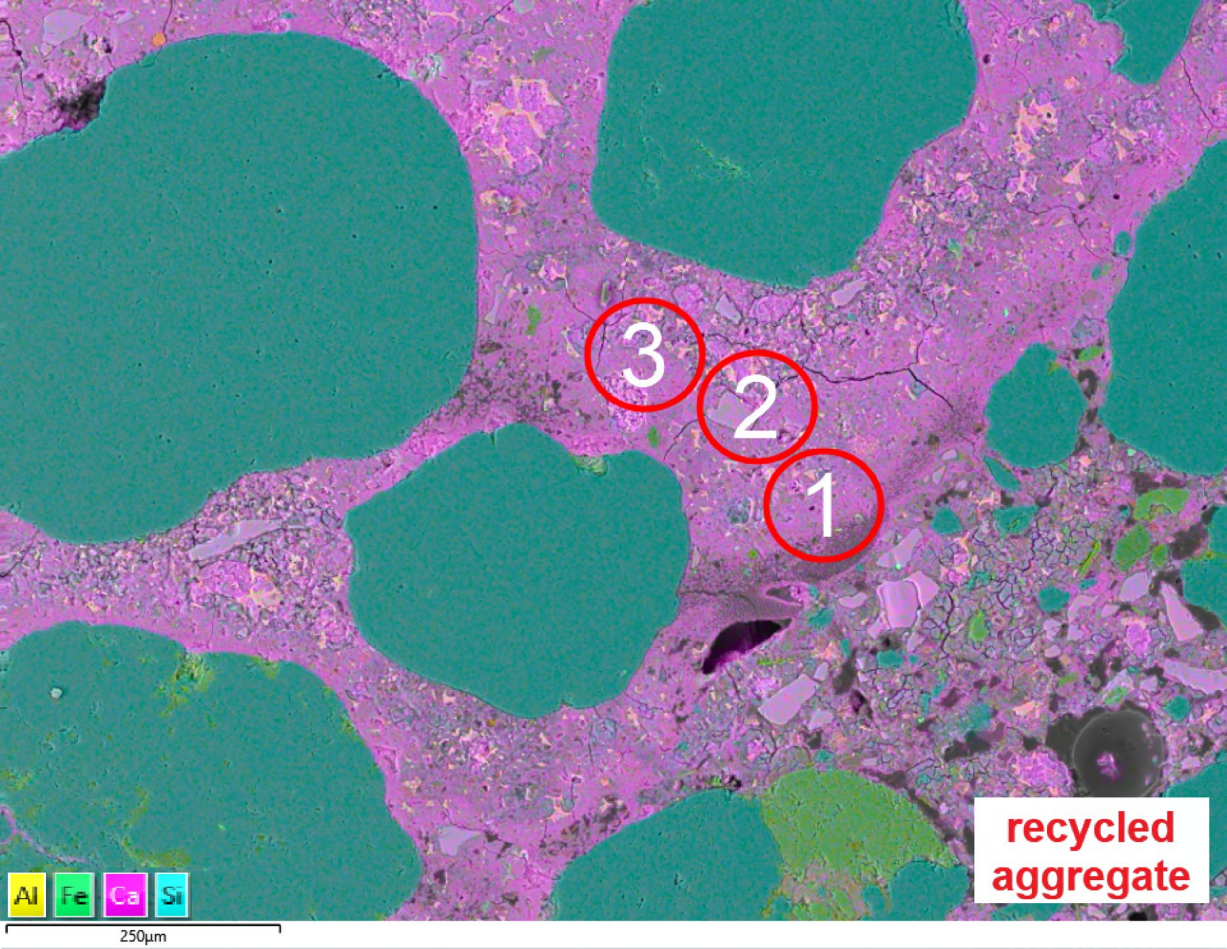
Example of analyzed areas of the cement matrix in the ST sample.

To perform a semi-quantitative assessment of the composition of the cement paste at various distances from the coarse aggregate-grout contact zone, SEM-EDX analyses of the elemental composition of three areas with a diameter of approximately 100 μm were performed. The areas were marked as 1, 2, and 3, starting from the contact zone, the medium zone, and the zone further from the coarse aggregate. Whenever possible, zones were also analyzed away from the fine aggregate grains. For each sample, three areas were examined under the same SEM exposure conditions (accelerating voltage, aperture, working distance, etc.). Average values were derived from the concentration (% at.) of the elements Ca, Si, Al, and Fe. The analysis focused on changes in calcium concentration as the most easily migrating ion in the cement matrix. An example of such analysis is presented in Fig. 9.

**Fig. 10:**
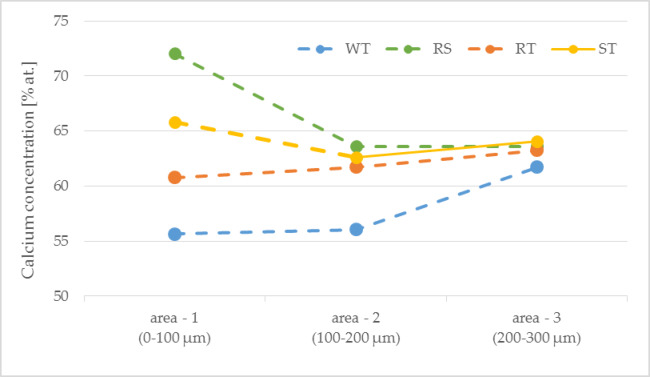
The concentration of calcium is a function of distance from the transition zone.

Figure 10 presents curves showing changes in the concentration of calcium ions depending on the distance from the transition zone of the paste-recycled aggregate.

The differences between samples have been spotted by analyzing the calcium concentration near the transition zone between the paste and recycled aggregates and its changes with increasing distance from this zone. The lowest concentration in area 1 (0–100 μm from the transition zone) was observed in WT samples, and it increases in other samples as follows: WT-RT-ST-RS. The concentration of calcium hydroxide mainly determines the concentration of calcium ions in the transition zone. The WT sample (Without Treatment) contains dry recycled aggregates containing crushing fines. Aggregate grains absorbed water from the concrete mix. As observed during SEM analysis, they were also covered with a thin layer of penalties, further increasing the absorption of water and portlandite. That explains why the concentration of portlandite in the transition zone in the WT sample is relatively low. It remains almost constant in area 2 (up to 200 μm) and increases in area 3 (200–300 μm). Analyzing the shape of the curve for the RT sample (Resin Treatment), it might be seen that the concentration of calcium ions in all areas remains almost constant, with a slight increase with increasing distance from the transition zone. This observation might be explained by the non-absorbing properties of recycled aggregates covered by the resin layer. As such, they do not influence the calcium concentration (portlandite) much in the areas of the cement matrix near the transition zone. A slight increase in the curve might be caused by a not fully covered surface of aggregates with the resin. The shapes of ST samples (Soake Treatment) and RS (Rinsed Soaked) curves are somewhat different than others. The concentration of portlandite in area 1 for both samples is higher than in other regions. In both samples, recycled aggregates were saturated with water before being added to the concrete mix. Although in ST samples aggregates contained fines from crushing, in RS samples, aggregates were previously rinsed with water to eliminate the fines. Water in the recycled aggregates grains migrates outside to the cement matrix, locally increasing the water-cement ratio and the concentration of portlandite, especially near the transition zone. However, fines in the ST samples might absorb some of the water with portlandite, which lowered the calcium concentration in the RS sample. In area 2, the calcium concentration in both samples decreases to the level measured for the RT sample and remains almost constant in area 3.

The calcium concentration in area 3 in all samples was almost equal, indicating that the water saturation of porous aggregate in the transition zone has its influence not more than 200–300 μm from the transition zone.

**Table 4:** Results of flexural and compressive strength tests and carbonation depth of analyzed concretes.

Composition	Admixture, % cement mass	Compressive strength, MPa		Flexural strength, MPa		Carbonation depth, mm	
		2 d	28 d	2 d	28 d	14 d	28 d
R	1.0	24.0 ± 0.1	49.2 ± 2.3	3.8 ± 0.2	5.9 ± 0.4	4.4	8.6
WT	3.0	19.2 ± 0.4	40.0 ± 0.8	2.5 ± 0.2	6.0 ± 0.4	7.5	7.5
RS	2.0	9.6 ± 0.6	35.8 ± 2.1	1.9 ± 0.1	5.3 ± 0.1	12.5	14.1
RT	1.0	12.7 ± 1.9	22.8 ± 0.4	2.0 ± 0.2	3.3 ± 0.1	0.1	0.1
ST	2.0	20.2 ± 0.3	49.1 ± 0.7	3.0 ± 0.1	5.8 ± 0.3	9.2	12.0

**Fig. 11:**
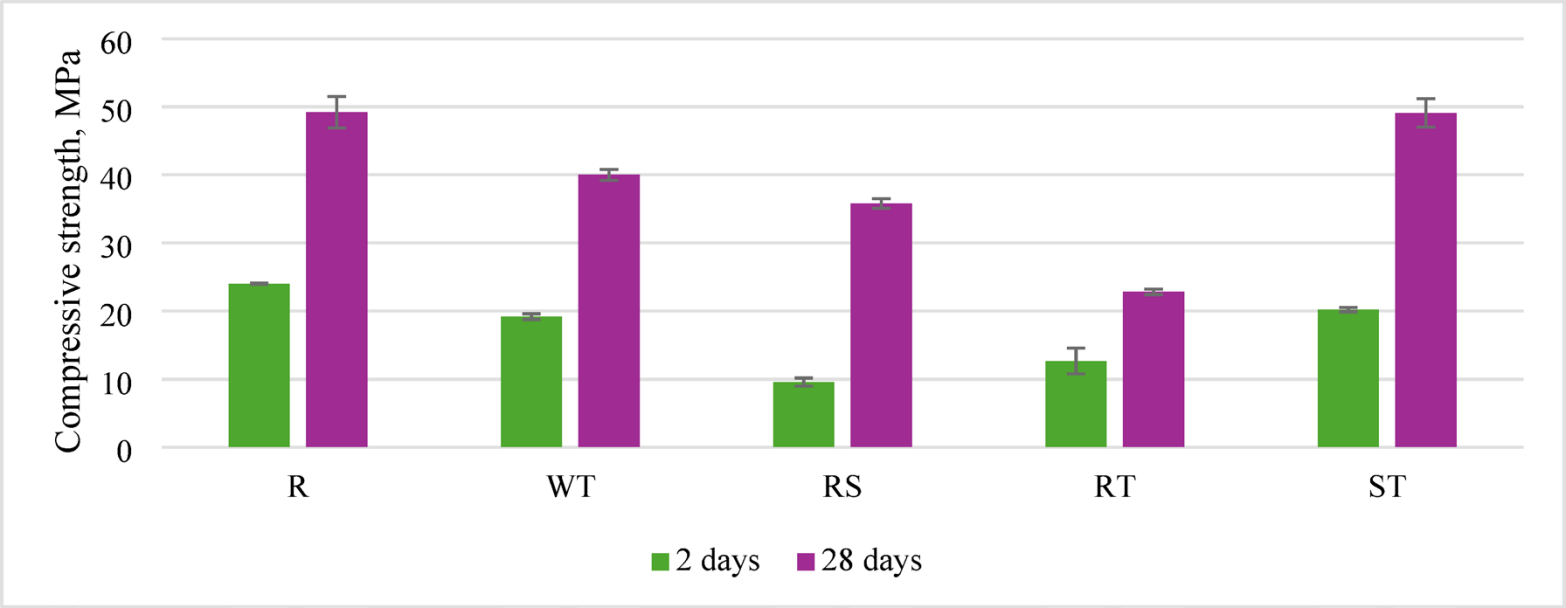
Compressive strength after 2 and 28 days of curing, depending on the preparation method of recycled aggregate.

The application of inadequately prepared recycled aggregate decreases compressive strength, observed at 2 and 28 days (Table 4). However, when recycled aggregate is ready through a washing process using a sieve, which removes fines and provides surface saturation, the differences in compressive strength compared to concrete without recycled aggregate fall within the standard deviation range of the results. A significant difference was noted when the preliminary preparation involved impregnating the surface of the aggregate grains with resin; due to inferior adhesion of the new cement paste to the grains, the compressive strength of concrete prepared this way is significantly lower (Fig. 11). Based on the presented analysis of the ITZ (Interfacial Transition Zone) area, it can be concluded that the sieve washing method allows for a high-quality ITZ, thereby contributing to high compressive strength in concrete.

**Fig. 12:**
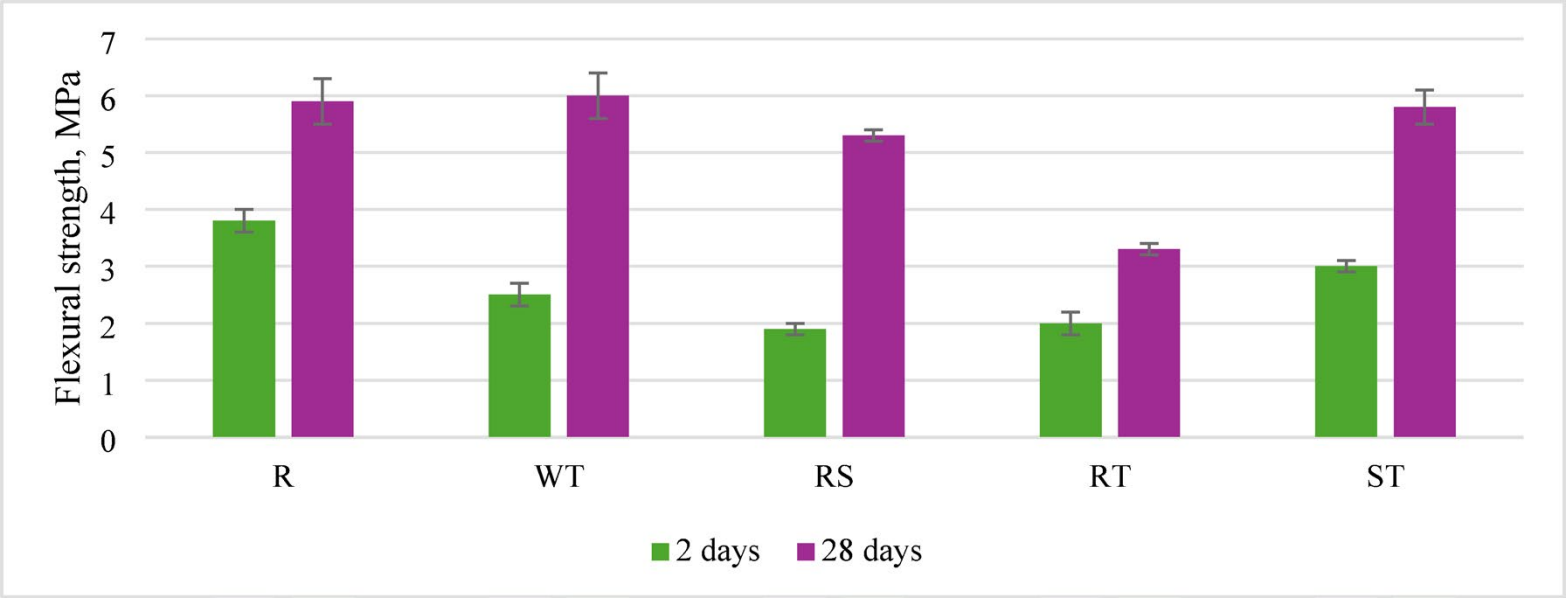
Flexural strength after 2 and 28 days of curing, depending on the preparation method of recycled aggregate.

This preparation method also results in significantly lower flexural strength than the other analyzed concretes. Different methods of preparing recycled aggregate did not considerably affect flexural strength, even compared to the reference concrete without recycled aggregate (Fig. 12).

While resin treatment showed superior carbonation resistance, SEM-EDX revealed a 2–5 μm interfacial gap between polymer coating and new cement paste (Fig. 8). This physical discontinuity, rather than chemical incompatibility, explains the 12% compressive strength reduction in RT specimens compared to untreated RA samples. Elemental mapping showed calcium silicate hydrate (C-S-H) formation continued up to the resin boundary, with Ca/Si ratios decreasing from 1.8 to 1.2 across the ITZ, indicating modified hydration dynamics rather than complete adhesion failure.

**Fig. 13:**
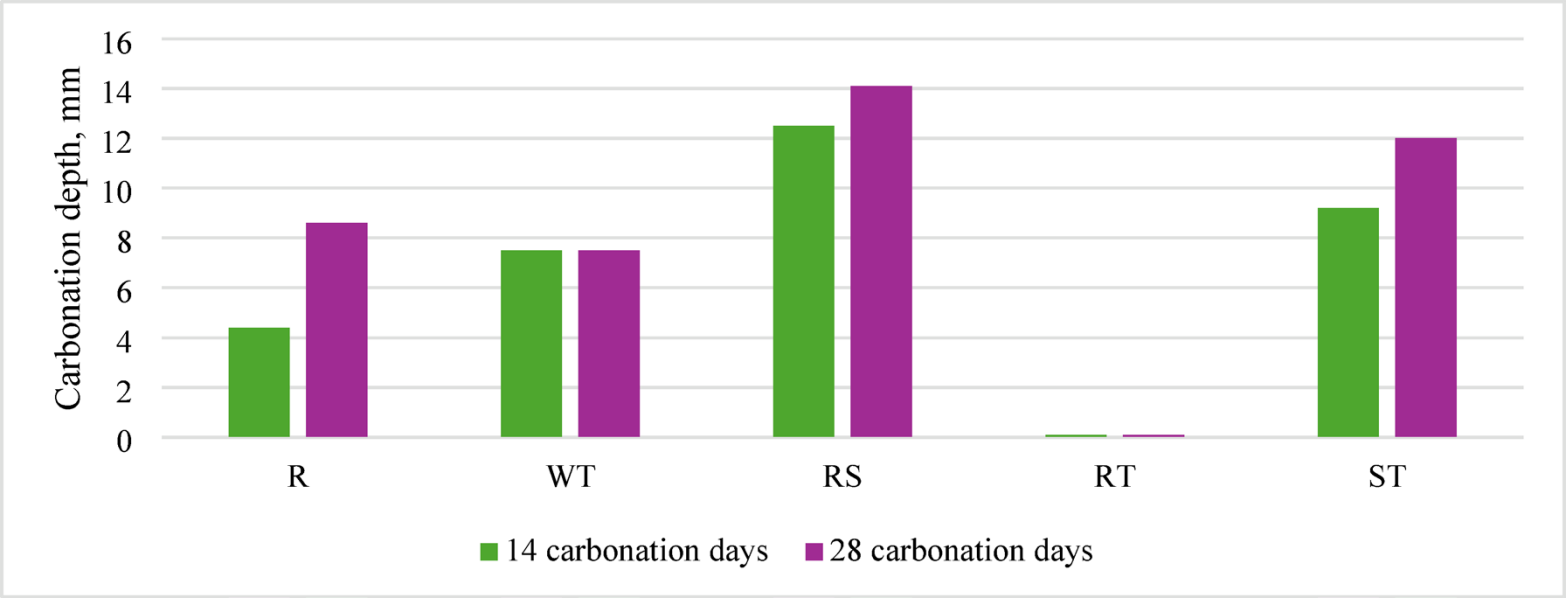
Carbonation depth after 14 and 28 days of accelerated carbonation, depending on the preparation method of recycled aggregate.

Concrete carbonation largely depends on porosity, including the ITZ area and the porous recycled aggregate grains. The obtained research results indicate that all the analyzed preparation methods have potential in this regard (Fig. 13).

**Fig. 14:**
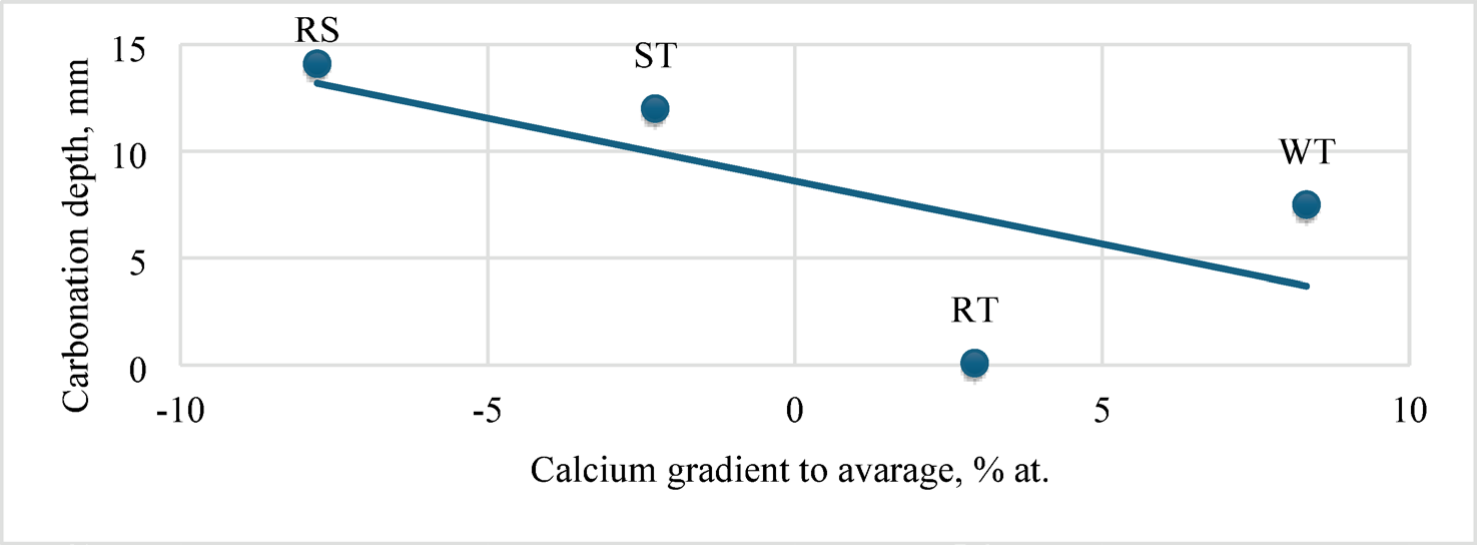
Carbonation depth after 28 days of accelerated carbonation, depending on the calcium gradient.

A notable inverse correlation was observed between the calcium gradient (relative to the average concentration in the area around 250 µ from the recycled aggregate surface) and carbonation depth. Specifically, higher calcium depletion near the aggregate surface, reflected by a steeper gradient, corresponded to reduced carbonation penetration. This suggests that a sharper gradient may indicate more efficient cement hydration and denser ITZ microstructure, which limits CO₂ ingress (Fig. 14).

## Conclusions

The analyzed concrete contained recycled aggregate with varying fines content, different levels of water saturation, and water absorption (resin-treated aggregates). Each of these factors individually influences the microstructure of the interfacial transition zone (ITZ). However, their combined effect ultimately determines the adhesion of the cement matrix to the aggregate surface, which is the primary factor governing the strength of the entire composite. As demonstrated by previous research^16,41^, the water saturation level of grains plays a crucial role in optimizing the effect of porous aggregates use in concrete. It significantly affects the microstructure of the aggregate-paste interface, particularly the portlandite content, which in turn influences the properties of the entire composite. Analyzing recycled aggregate concrete without considering the pre-treatment method is insufficient^42^.

This study systematically evaluated the effects of different recycled aggregate pretreatment methods on the microstructure, mechanical properties, and carbonation resistance of recycled aggregate concrete (RAC). The key conclusions are as follows:

- Inverse Correlation Between Calcium Gradient and Carbonation Depth: A significant inverse correlation was observed between the calcium ion concentration gradient near the recycled aggregate surface and carbonation depth. Samples exhibiting a steeper calcium depletion gradient—indicative of more efficient cement hydration and a denser interfacial transition zone (ITZ)—demonstrated markedly reduced carbonation penetration. This finding highlights the calcium gradient as a valuable microstructural indicator of ITZ quality and durability performance.
- Mechanical Performance and Pretreatment Effects: Incorporating recycled aggregates generally resulted in reduced compressive and flexural strengths compared to natural aggregate concrete. Water immersion and sieve-washing improved mechanical strength among the pretreatment methods, achieving values comparable to the reference concrete after 28 days. Resin impregnation, while significantly enhancing carbonation resistance by drastically limiting CO₂ ingress, caused a moderate decrease in compressive strength, likely due to the formation of a thin interfacial layer affecting bond quality.
- Trade-Off Between Strength and Durability: The results demonstrate a trade-off between mechanical strength and durability enhancement. Resin impregnation offers superior durability benefits, particularly in reducing carbonation depth, but at the expense of some strength loss. Conversely, water-based pretreatments provide a balanced improvement in both strength and durability.
- Practical Implications for RAC Production: The findings suggest that selecting appropriate pretreatment methods can optimize RAC properties according to specific application requirements. For structures where carbonation resistance is critical, resin impregnation is recommended despite its moderate strength reduction. Water immersion or sieve-washing offers practical and economical solutions for applications prioritizing mechanical performance.
- Water immersion pretreatment emerged as the most balanced approach for achieving optimal mechanical properties. After 28 days of curing, concrete with saturated recycled aggregate demonstrated compressive strength of 49.1 ± 0.7 MPa and flexural strength of 5.8 ± 0.3 MPa, closely approximating those achieved with natural aggregate concrete. This performance enhancement is attributed to optimizing moisture release during hydration, facilitating improved ITZ formation between the recycled aggregate and the new cement matrix. The 24-hour soaking protocol ensures that approximately 50% of the absorbed water participates in the effective water-cement ratio, as evidenced by rheological observations showing equivalent slump values when total water content was reduced by 48–52%.

In summary, this research advances the understanding of the microstructural mechanisms governing RAC durability and strength, providing a predictive basis for improving recycled aggregate concrete through tailored pretreatment strategies.

## Data Availability

The datasets used and/or analysed during the current study available from the corresponding author on reasonable request.
